# Effects of superfine grinding sweet potato leaf powders on physicochemical and structure properties of sweet potato starch noodles

**DOI:** 10.1002/fsn3.3593

**Published:** 2023-08-02

**Authors:** Guanghui Li, Xueli Gao, Yonghui Wang, Shenghua He, Weiyun Guo, Jihong Huang

**Affiliations:** ^1^ Food and Pharmacy College Xuchang University Xuchang China; ^2^ College of Agriculture Henan University Zhengzhou China

**Keywords:** cooking characteristics, FTIR, starch noodles, superfine grinding, sweet potato leaves, XRD

## Abstract

Sweet potato leaves (SPLs) containing abundant functional components are consumed primarily as fresh vegetables worldwide. This study investigated the physical properties of superfine grinding SPLs powder, and their effects on cooking, texture, and sensory properties, micro‐ and molecular structures of starch noodles were also explored. The results showed that the bulk and tapped density (from 0.34 to 0.28 g/mL^3^ and from 0.69 to 0.61 g/mL^3^), repose and slid angle (from 42.15 to 30.96° and from 48.67 to 22.00°), water‐holding capacity and swelling capacity (from 8.66 to 4.94 g/g and from 10.03 to 7.77 mL/g) of SPLs powders were decreased with milling time increased. The cooking loss, swelling index, texture, and sensory properties of SPLs sweet potato starch noodles (SPLSNs) were improved as the particle size of SPLs decreased. XRD and FT‐IR showed that SPLSNs contained less complete crystallites (from 28.85% to 14.19%) and lower proportion of crystalline region (*R*_1047/1017_ from 0.96 to 0.81, *R*_1017/994_ from 0.41 to 0.43). SEM revealed that SPLSNs exhibited fewer ordered arrays and smooth cross sections. Our findings provide a foundation for utilizing SPLs and developing functional starch noodles.

## Graphical abstract

SPLSNs contain less complete crystallites.

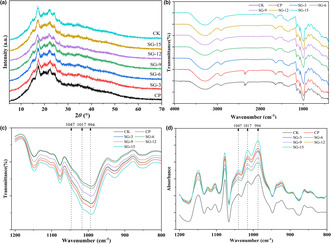

## 1 INTRODUCTION

Sweet potato starch noodle (SPSN), made from sweet potato starch only, is one of the most popular traditional foods in Asia, especially in China. SPSN is suitable for a gluten‐free diet because it does not contain wheat starch. However, SPSN is deficient in protein, dietary fiber, vitamins, etc. With the demand for healthy, nutritious, convenient, and safe foods, starch noodles containing ingredients such as quinoa, yellow lentil, red lentil protein, potato residue, curcumin, tea polyphenols, and lycopene have been reported (Geng et al., 2021; Guo et al., 2022; Huang et al., 2022; Odabas & Cakmak, 2022).

Sweet potato leaves (SPLs) are the aboveground parts of the sweet potato during its growth, with a total yield of about 100 million tons annually in China. As one of China's traditional spices and herbs, SPLs contain functional compounds such as chlorogenic acids, carotenoids, flavonoids, and caffeoylquinic derivatives and therefore have a wide variety of functions, including antidiabetic, antimutagenic, antioxidant, antimicrobial, and anticarcinogenic (Nguyen et al., 2021; Sun et al., 2014). However, only a few SPLs are used as vegetables for humans or as feed for animals, most are merely discarded, leading to serious resource waste and environmental pollution. Therefore, effective measures should be taken to improve the utilization of the SPLs and promote the sustainable development of the sweet potato industry.

Superfine grinding, as a novel technique, is an efficient tool for making superfine powder. The superfine powders (approximately 10–30 μm in size) exhibit excellent characteristics, such as better surface properties, stronger solubility and fluidity, better flavor release, and mouthfeel (Waliullah et al., 2021). This technology has been commonly applied in the food industry, and the superfine powders of *Sargassum fusiforme* residue (SFR), red grape pomace, *Vaccinium bracteatum Thunb* leaves, and Sanchi (*Panax notoginseng*) flower have been reported (Gan et al., 2022; Jiang et al., 2017; Wu et al., 2020; Zhao et al., 2015). These superfine powders exhibit improved physicochemical properties compared to conventional powders. Applying superfine ingredients to bakery products has been extensively reported (Gan et al., 2022; Kim et al., 2023). Gan et al. (2022) found that as the amounts of superfine grinding SFR powder increased, the hardness and chewiness of sponge cake and the density and viscosity of cake batter increased. According to Kim et al. (2023), as the marigold powder's particle size decreased, the sponge cake's specific volume increased, whereas baking loss, hardness, and chewiness decreased. Gan et al. (2023) also reported that the smaller the particle size of SFR powder, the lower the lightness and yellowness, and the higher the spread ratio of cookies.

The chemical composition (such as carotenoids, chlorogenic acid, and quercetin) and health functionalities (including antioxidation, antidiabetics, anticancer, anti‐inflammation, and antibacteria) of SPLs have been extensively reported (Nguyen et al., 2021). However, limited knowledge is available for the properties of superfine SPLs powder and SPLs‐based starch noodles. Therefore, this study aimed to evaluate the physicochemical properties of SPLs powders prepared by superfine grinding. In addition, changes in the qualities (cooking, texture, and sensory properties) of the SPSN containing different SPLs powders were discussed. Finally, the mechanism of SPLs powder on SPSN was determined by Fourier transform infrared spectroscopy (FI‐IR), X‐ray diffraction (XRD), and scanning electron microscopy (SEM).

## 2 MATERIALS AND METHODS

### 2.1 Materials

Sweet potato starch (SPS) containing 27.5% amylose was obtained from Shandong Shengqi Biological Co., Ltd. SPLs of Shangshu‐19 were harvested at the fully grown stage (in October 2021) by Henan Shengtian AGRICULTURE Co., Ltd.

### 2.2 Preparation of SPLs powders

The wet SPLs were dried at 55°C for 24 h in an air‐drying oven, and the water content of the dried SPLs was less than 10%. The samples were ground coarsely for 2 min using a multifunction pulverizer (Sufeng Industry & Trade Co., Ltd.). A sieve with a mesh size of 60 was used to screen the particles. Then, the powders were processed in an NLD‐6DI micronizer from Nalide Superfine Grinding Technology Co., Ltd. for 3, 6, 9, 12, and 15 min; finally, five different particle sizes of SPLs were obtained, named M_3_, M_6_, M_9_, M_12_, and M_15_, respectively. The coarse powder (that passed through a sieve of 60 mesh) was used as CK.

### 2.3 Determination of the particle size distribution

The Mastersizer 2000 (Malvern Instruments Co., Ltd.) was used to measure the particle size distribution of SPLs powder. Before measurement, double‐distilled water was used to mix the SPLs powder. *D*_0.1_, *D*_0.5_, and *D*_0.9_ values are generally taken to represent the particle size distribution. The span, which represents the width of the particle size, is calculated by the following equation:

(1) $$\text{Span}=\left(D_{0.9}-D_{0.1}\right)/D_{0.5}$$

### 2.4 Determination of the repose and slide angle

According to the methods described by Li et al. (2020) and Huang et al. (2018), the repose angle (*θ*) was measured as follows. In brief, the funnel was fastened to the retort stand and placed vertically above the graph paper. Then, the SPLs powder was poured into the funnel until the powder cone's peak just touched the funnel's outlet. The cone's height (*H*) and diameter (*R*) were determined, and the equation below was used to compute the repose angle:

(2) $$\theta =\text{arctg}\;\left(R/H\right)$$

The slide angle (*α*) was determined using the method of Huang et al. (2018) with minor modifications. A glass plane was covered with SPLs powder (5.0 g), which was then agitated enough to disperse evenly. The glass plane was then raised until the SPLs powder began to slip. The slide angle was determined by the relationship between the horizontal plane and the inclined glass plane. Both the length of the glass plane (*L*) and the vertical distance (*h*) between the inclined glass plane's top and horizontal plane were measured. The following equation was used to get the slide angle:

(3) $$\alpha =\arcsin\;\left(h/L\right)$$

### 2.5 Determination of the bulk and tapped density

The method described by Li et al. (2020) was used to determine the bulk and tapped density.

For the bulk density, the SPLs powder was added to the 10‐mL volumetric flask (m_1_) and then weighed (m_2_). The following formula was used to get the SPLs powder's bulk density:

(4) $$\rho_{\text{bulk}}=\left(m_{2}-m_{1}\right)/10$$

The tapped density was determined as follows: SPLs powder (*M*, 10.0 g) was precisely weighed and added to the graduated measuring glass cylinder (10 mL). The cylinder was then gently shocked on a thick rubber blanket until the powder's volume was no longer lowered. The powder's final volume was recorded as *V* (mL). The equation below was used to calculate the tapped density:

(5) $$\rho_{\mathrm{tap}}=M/V$$

### 2.6 Determination of water‐holding, oil‐binding and swelling capacities

Water‐holding capacity (WHC) was measured as described by Gan et al. (2022) with minor modifications. SPLs powder (m_1_, 0.5 g) and distilled water (25 mL) were poured into the centrifuge tube (m_2_) and then evenly mixed by a vortex. The tube was kept in a water bath (50°C, 30 min) and then filtered. The filtrate was abandoned, and the tube with wet powder (m_3_) was weighed. The following equation was used to calculate the WHC:

(6) $$\mathrm{WHC}=\left(m_{3}-m_{2}-m_{1}\right)/m_{1}$$

The method of Li et al. (2020) was used to assess the oil‐binding capacity (OBC). A quantity of 25 mL of soybean oil and 0.5 g of the powder (m_0_) were added to the centrifuge tube (m_1_). The mixture was centrifuged (1600 *g*, 10 min) after the tube was held in a water bath (30°C, 30 min). After discarding the supernatant, the weight of the tube containing the wet powder (m_2_) was calculated. The following equation was used to determine the OBC:

(6) $$\mathrm{OBC}=\left(m_{2}-m_{1}-m_{0}\right)/m_{0}$$

Swelling capacity (SC) was measured using the method of Gan et al. (2022). In a graduated cylinder, 1 g of the powder (*M*) was mixed with 25 mL of distilled water. After 24 h, the powder's addition volume (*V*_1_) after hydration was measured. The SC was calculated using the following equation:

(7) $$\mathrm{SC}=V_{1}/M$$

### 2.7 Determination of the water solubility index

The water solubility index (WSI) was measured using the method described by Jiang et al. (2017) with slight modifications. Briefly, a total of 1.0 g of powder and 50‐mL distilled water were mixed in a centrifuge tube and then placed in a water bath (55°C) for 30 min. Subsequently, the tube was centrifuged (1700 *g*, 10 min). The supernatant was dried (100°C) for 12 h before determining the weight of dry powder (*A*). The WSI was calculated using the following formula:

(8) $$\mathrm{WSI}\;\left(\%\right)=A\times 100\%$$

### 2.8 Preparation of starch noodles

According to the method of Li et al. (2019) with modifications, the process of SPLs sweet potato starch noodles (SPLSNs) was as follows: The sweet potato starch (80 g) and distilled water (85.0 mL) were mixed evenly, and the mixture was gelatinized in a water bath (100°C) to obtain starch paste. The paste was then combined to create the starch dough by adding the sweet potato starch (15.5 g) and SPLs powder (4.5 g). The starch dough was wrapped in plastic and left to rest for 10 min at 25°C. Finally, the dough was extruded using a pressure noodle machine with a 2.50 mm diameter mold. The wet starch noodles were about 10 cm in length. After boiling for 2 min, the noodles were allowed to chill in distilled water for 1 min. The SPLSNs were dried at room temperature (approximately 20°C) for 48 h before being placed in plastic bags for storage. SPSN without SPLs was used as control, and SPSN with coarse powder, M_3_, M_6_, M_9_, M_12_, and M_15_ were expressed as CP, SG‐3, SG‐6, SG‐9, SG‐12, and SG‐15, respectively.

### 2.9 Cooking properties of the SPLSNs

The ratio of broken noodles (RBN) was determined as follows: 20 pieces of SPLSNs (each 10 cm long) were boiled in hot water for 10 min. After removing the noodles from the boiling water, the total number of broken noodles was counted. The RBN was calculated as the ratio of broken cooked noodles to uncooked noodles.

The cooking loss (CL) and swelling index (SI) of the SPLSNs were evaluated by the method of Menon et al. (2016) with minor modifications. In 100 mL of boiling water, SPLSNs (*M*_0_, 3.0 g) were cooked for 15 min with minimal stirring. The cooked SPLSNs were collected, drained of surface water using filter paper, and promptly weighed (*M*_1_). The cooked noodles were then dried at 105°C to a consistent weight (*M*_2_). The following formulas were used to determine the CL (%) and SI:

(9) $$\mathrm{CL}=\left(M_{0}-M_{2}\right)/M_{0}\times 100$$

(10) $$\mathrm{SI}=\left(M_{1}-M_{2}\right)/M_{2}$$

### 2.10 Sensory analysis of the cooked SPLSNs

Sensory attributes of SPLSNs were evaluated by 25 food science students, including 13 females and 12 males aged 19–24 years from Xuchang University. A quantity of 250 g of dried SPLSNs was cooked in boiling water for 5 min and then packed in individual plastic bags, which were labeled with random numbers. The panel members were guided on how to carry out the tests. The panelists scored the attributes, including color (total of 20 points), appearance (total of 30 points), texture (total of 20 points), and mouth feel (total of 30 points).

### 2.11 Texture analysis

SPLSNs (15 g) were cooked for 5 min in boiling water (300 mL), followed by a 15‐s washing in distilled water. Plastic wrap was used for packaging the cooked SPLSNs until further use. The textural properties of cooked SPLSNs were assessed using the texture analyzer (TMS‐PRO, Food Technology Corporation) within 10 min. Using a 50‐mm flat probe and a test speed of 6.0 mm/min, three parallel strands of cooked SPLSNs were crushed crosswise twice to 80% of their original height. The texture analyzer software analyzed the hardness, adhesiveness, springiness, cohesiveness, gumminess, and chewiness based on TPA force‐time curves (Yadav et al., 2014).

### 2.12 Determination of the morphology

Scanning electron microscopy (SEM; Gemini SEM360, Zeiss) was used to examine the microstructures of the dried SPLSNs' cross section at a magnification of 100 and an operating voltage of 15 kV.

### 2.13 X‐ray diffraction (XRD) analysis

The X‐ray diffractometer (Bruker AXS Inc.), equipped with Cu radiation at a wavelength of 1.5406 Å, was used to determine the long‐range ordered structures of SPLSNs. The dried SPLSNs were ground and passed through a 60‐mesh sieve. The diffraction 2θ was 4–60°, and measurements were made at 25°C with a scanning rate of 2°/min. The percentage of crystalline area relative to the total diffraction area was defined as the relative crystallinity (%; Xiang et al., 2018).

### 2.14 Analysis of FT‐IR

The Nicolet IS50 (Thermo Scientific) was used to collect FT‐IR spectra of SPLSNs. With 64 scans at a resolution of 4 cm^−1^, the FT‐IR spectra were recorded between 400 and 4000 cm^−1^. The FT‐IR data were analyzed between 800 and 1200 cm^−1^. Following baseline correction, the spectra were deconvolved with a half‐width of 20.0 cm^−1^ and a resolution enhancement factor of 2.0. In order to investigate the short‐range ordered structure of SPLSNs samples, the ratios of FT‐IR absorbance at 1047 to 1017 cm^−1^ (coded as *R*_1047/1017_) and 1017 to 994 cm^−1^ (coded as *R*_1017/994_) were calculated (Yang, Dhital, et al., 2021).

### 2.15 Statistical analysis

All tests were carried out in triplicate, and the results were reported as mean ± standard deviation (SD). One‐way ANOVA (Duncan's test) was used to analyze the significant differences, and *p* < .05 was considered statistically significant. All plotted data were processed by Origin 2021.

## 3 RESULTS AND DISCUSSION

### 3.1 Particle size distributions of SPLs powder

Table 1 shows the particle size distribution of the SPLs powder. The D_10_, D_50_, D_90_, D(3,2), and D(4,3) are used to characterize the particle size distributions. D_10_, D_50,_ and D_90_ significantly decreased as grinding time increased from 36.60 to 7.00 μm, 337.70 to 26.11 μm, and 814.90 to 71.18 μm, respectively. D_50_, the average median diameter, is used as an indicator of the cohesiveness of the powder. The D_50_ of various SPLs powders were as follows: 337.70, 110.32, 73.03, 66.00, 47.23, and 26.11 μm, respectively. This demonstrated that a vibration mill could produce superfine SPLs powder. Jiang et al. (2017) reported similar results that the superfine powders of *Vaccinium bracteatum Thunb* leaves with particle sizes of 22.03 and 13.73 μm were produced using a micromilling process.

**TABLE 1:** Particle size distributions of sweet potato leaves powder.

Samples	CK	M_3_	M_6_	M_9_	M_12_	M_15_
D_10_ (μm)	36.60 ± 1.51^a^	13.18 ± 0.31^b^	10.76 ± 0.36^c^	10.85 ± 0.39^c^	9.28 ± 0.26^c^	7.00 ± 0.12^d^
D_50_ (μm)	337.70 ± 6.03^a^	110.32 ± 1.30^b^	73.03 ± 2.02^c^	66.00 ± 1.53^d^	47.23 ± 0.99^e^	26.11 ± 0.50^f^
D_90_ (μm)	814.90 ± 40.56^a^	389.59 ± 0.17^b^	240.39 ± 3.89^c^	195.05 ± 2.62^d^	145.34 ± 4.23^e^	71.18 ± 2.77^f^
D(3,2) (μm)	60.37 ± 2.28^a^	26.90 ± 0.40^b^	22.22 ± 0.55^c^	21.30 ± 0.65^c^	21.49 ± 0.36^c^	18.53 ± 0.15^d^
D(4,3) (μm)	385.86 ± 15.04^a^	161.82 ± 0.72^b^	103.13 ± 2.03^c^	77.02 ± 0.86^d^	60.95 ± 1.7^d^	49.79 ± 2.13^e^
Span	2.30 ± 0.06^e^	3.41 ± 0.04^a^	3.14 ± 0.04^b^	2.96 ± 0.10^c^	2.79 ± 0.03^d^	1.83 ± 0.01^f^
Specific surface area (m^2^/g)	0.10 ± 0.00^d^	0.22 ± 0.00^c^	0.27 ± 0.01^b^	0.28 ± 0.01^b^	0.28 ± 0.00^b^	0.32 ± 0.00^a^

*Note*: Mean values in the same row with different letters are significantly different (*p* < .05).

The surface characteristics of the SPLs powder are listed in Table 1. The specific surface area increased from 0.10 to 0.32 m^2^/g as the particle size decreased, but the surface–number mean (D(3,2)) and volume–surface mean (D(4,3)) declined from 60.37 to 18.53 μm and 385.86 to 49.79 μm, respectively. The surface properties of SPLs powder varied significantly between different SPLs powders (*p* < .05). The SPLs powder with a particle size of 26.11 μm had a higher specific surface area value (0.32 m^2^/g) and lower D(3,2) and D(4,3) values (18.53 and 49.79 μm) due to the superfine grinding. The specific surface area of ultrafine powder increased was related to the more particles per unit weight. When combined with additional powders, superfine powders have a higher tendency to achieve homogeneity. If the SPLs powder had more surface area, the active functional components could dissolve faster, and the powder would be more bioavailable and absorbable. These findings for SPLs powder were consistent with those for soybean residue and Dendrobium officinale (Li et al., 2020; Meng et al., 2017).

The “span” denotes the particle size distribution's width. A small span value means that the size distribution of the particles is narrow and their sizes are more uniform. With increasing milling time, the span values first increased (from 2.30 to 3.41) and subsequently decreased (from 3.14 to 1.83). This result disagreed with Li et al. (2020), who found that as the size decreased, the span values of the soybean residue powder decreased first and then increased. The difference could be due to the grain size, the material, or the grinding process.

### 3.2 Physicochemical properties of SPLs powder

The repose and slide angles of SPLs powder are shown in Table 2. As the milling time increased, the repose and slide angles decreased. The repose and slide angles ranged from 42.15° to 30.96° and 48.67° to 22.00°, respectively. There were differences in the angle of repose or slide among the SPLs powder (*p* < .05). This may be due to the formation of aggregates, which tended to arrange in a cone. Among these powders, superfine powder M_15_ had the lowest angle of repose and slide (30.96° and 22.00°). Others were M_12_ (31.19° and 29.33°), M_9_ (31.66° and 31.83°), M_6_ (33.02° and 35.33°), M_3_ (35.82° and 42.33°), and CK (42.15° and 48.67°). The fluidity of the SPLs powder improved as the repose and slide angles decreased. The M_15_ exhibited prominent fluidity and surface attachment. The findings were consistent with those of the studies published by Chen et al. (2015) and Zhao et al. (2009).

**TABLE 2:** Physicochemical properties of sweet potato leaves powder.

Samples	CK	M_3_	M_6_	M_9_	M_12_	M_15_
Angle of repose (°)	42.15 ± 1.20^a^	35.82 ± 1.31^b^	33.02 ± 0.67^c^	31.66 ± 0.70^cd^	31.19 ± 1.06^d^	30.96 ± 0.71^d^
Angle of slide (°)	48.67 ± 0.58^a^	42.33 ± 1.53^b^	35.33 ± 0.58^c^	31.83 ± 0.76^d^	29.33 ± 1.53^e^	22.00 ± 1.00^f^
Bulk density of powder (g/mL)	0.34 ± 0.00^a^	0.31 ± 0.00^b^	0.30 ± 0.00^c^	0.30 ± 0.01^c^	0.28 ± 0.00^d^	0.28 ± 0.00^e^
Tapped density of powder (g/mL)	0.69 ± 0.01^a^	0.67 ± 0.01^b^	0.66 ± 0.01^c^	0.64 ± 0.00^d^	0.63 ± 0.01^d^	0.61 ± 0.01^e^
Water‐holding capacity (g/g)	8.66 ± 0.34^a^	5.76 ± 0.18^b^	5.20 ± 0.05^c^	4.81 ± 0.29^c^	4.93 ± 0.10^c^	4.94 ± 0.17^c^
Oil‐binding capacity (g/g)	2.75 ± 0.11^a^	2.26 ± 0.02^b^	2.14 ± 0.05^b^	1.99 ± 0.07^c^	1.91 ± 0.08^c^	1.93 ± 0.06^c^
Water solubility index (%)	31.33 ± 0.68^c^	32.42 ± 0.28^b^	33.58 ± 0.03^a^	33.66 ± 0.39^a^	33.69 ± 0.02^a^	33.90 ± 0.03^a^
Swelling capacity (mL/g)	10.03 ± 0.05^a^	9.83 ± 0.21^ab^	9.43 ± 0.45^b^	8.92 ± 0.33^c^	8.85 ± 0.23^c^	7.77 ± 0.21^d^

*Note*: Mean values in the same row with different letters are significantly different (*p* < .05).

With increased milling time, the bulk and tapped densities were reduced. The bulk and tapped densities of SPLs powder were 0.34 to 0.28 g/mL and 0.69 to 0.61 g/mL, respectively (Table 2). SPLs superfine powders had significantly lower bulk and tapped densities than those produced by regular grinding (CK). The results indicated that the interparticle voids of superfine powder were larger, resulting in a larger contact surface with the surroundings. Our findings were consistent with those of Zhao et al. (2015) and Hu et al. (2012). However, the results disagreed with Huang et al. (2018) and Jiang et al. (2017). Huang et al. (2018) demonstrated that as particle size decreased, the bulk and tap density densities (from 0.453 to 0.620 × 10^3^ kg/m^3^, from 0.487 to 0.658 × 10^3^ kg/m^3^) increased. Jiang et al. (2017) proved that the tapped density of VBTL powders increased (from 0.451 to 0.613 g/mL) as the particle size decreased. This difference may be due to the particle's characteristics.

Table 2 lists the WHC, OBC, WSI, and SC. OBC was reduced from 2.75 to 1.93 g/g after superfine grinding. This may be due to the fact that superfine grinding damaged the structure of dietary fiber in SPLs. As the increase of milling time, WSI significantly increased from 31.33 to 33.90 g/g, indicating that a particle size decrease can considerably increase powder's solubility. This result consisted of previous studies (Gan et al., 2022; Jiang et al., 2017; Zhong et al., 2016). After superfine grinding treatment, WHC and SC were dramatically reduced from 8.66 to 4.94 g/g and 10.03 to 7.77 g/g, respectively. The porous matrix structure created by the fiber may impact WHC and SC, which represent a powder's capacity to retain moisture and swell. WHC and SC were reduced as a result of the porous matrix structure being broken by the superfine grinding. Gan et al. (2022) reported that WHC and SC were reduced from 5.82 to 3.15 g/g and 4.77 to 2.54 g/g after superfine grinding. Zhu et al. (2010) found that WHC and SC of wheat bran dietary fiber powder decreased (from 5.89 to 3.45 g/g and from 5.79 to 4.65 g/g) following superfine grinding. However, some studies, such as Huang et al. (2018), Meng et al. (2017), and Zhao et al. (2009), found the opposite results. The increase in surface area and total pore volume may contribute to the rise in hydration qualities, whereas the breakdown of the fiber matrix and collapse of pores during grinding may contribute to the decrease in hydration properties. Therefore, the modification of hydration properties is highly dependent on the types of raw materials, how they are processed, and the instrument parameters (Sangnark & Noomhorm, 2003).

### 3.3 Cooking quality of SPLSNs

Cooking quality is used to assess the acceptability of noodles by consumers. Noodles with low cooking loss, excellent surface conditions, and low stickiness are considered better. Both CL and SI contribute to the cooking quality, and the results for these parameters are shown in Table 3. With the increase in the milling time, the CL and SI of SPLSNs reduced significantly from 0.23% to 0.14% and from 3.70% to 3.09%. The Chinese Agriculture Trade Standards consider a CL during cooking of less than 10% acceptable, and the CL of SPLSNs was below 0.23% in the present study. This was in accordance with Odabas and Cakmak (2022), who found that quinoa and yellow lentil flour increased CL of gluten‐free noodles, but all noodles fell within acceptable limits. The smaller the particle size of SPLs powder, the lower CL of SPLSNs. This may be because that SPLs powder with smaller particles made the starch noodles' structure tighter. The SI of the SG‐6, SG‐9, SG‐12, and SG‐15 samples were lower than those of the SG‐3 and CP samples but greater than those of the CK. The SI of SPLSNs was positively related to the SI of SPLs powders. It was suggested that the SPLs powder with different particle sizes have various water‐holding capacities. The RBN was zero for all treatments.

**TABLE 3:** Cooking, sensory and textural properties of SPLs sweet potato starch noodles.

Parameter	CK	CP	SG‐3	SG‐6	SG‐9	SG‐12	SG‐15
Cooking properties							
Ratio of broken noodles (%)	0	0	0	0	0	0	0
Cooking loss (%)	0.14 ± 0.01^c^	0.23 ± 0.00^a^	0.18 ± 0.00^b^	0.15 ± 0.01^c^	0.15 ± 0.00^c^	0.15 ± 0.00^c^	0.14 ± 0.01^c^
Swelling index	2.35 ± 0.02^d^	3.70 ± 0.02^a^	3.41 ± 0.06^b^	3.03 ± 0.12^c^	3.00 ± 0.01^c^	3.03 ± 0.02^c^	3.09 ± 0.01^c^
Sensory properties							
Appearance	27.60 ± 0.45^a^	27.80 ± 0.45^a^	27.40 ± 0.55^a^	27.60 ± 0.55^a^	27.80 ± 0.45^a^	27.40 ± 0.55^a^	27.40 ± 0.55^a^
Color	16.80 ± 0.44^a^	15.20 ± 0.84^d^	16.00 ± 1.00^cd^	16.20 ± 0.84^bcd^	17.20 ± 0.84^a^	17.40 ± 0.55^a^	17.40 ± 0.55^a^
Texture	17.20 ± 0.45^a^	16.40 ± 0.89^b^	17.60 ± 0.55^a^	17.80 ± 0.45^a^	17.81 ± 0.45^a^	17.60 ± 0.55^a^	18.00 ± 0.71^a^
Mouth feel	27.60 ± 0.55^a^	26.80 ± 0.84^b^	27.40 ± 0.55^ab^	27.60 ± 0.55^a^	27.80 ± 0.45^a^	27.81 ± 0.35^a^	27.90 ± 0.45^a^
Textural properties							
Hardness (*N*)	88.35 ± 2.90^a^	30.10 ± 0.14^f^	45.00 ± 4.67^e^	52.95 ± 0.92^d^	53.10 ± 0.14^d^	64.55 ± 3.18^c^	81.65 ± 1.34^b^
Cohesiveness (Ratio)	0.37 ± 0.02^c^	0.53 ± 0.03^a^	0.55 ± 0.03^a^	0.47 ± 0.05^b^	0.38 ± 0.01^c^	0.36 ± 0.03^c^	0.30 ± 0.03^d^
Springiness (mm)	1.70 ± 0.06^b^	4.00 ± 0.01^a^	1.54 ± 0.15^c^	1.37 ± 0.07^d^	1.34 ± 0.06^d^	1.36 ± 0.04^d^	1.38 ± 0.02^d^
Gumminess (*N*)	32.75 ± 1.20^a^	16.40 ± 1.25^c^	22.60 ± 2.85^b^	22.63 ± 0.40^b^	22.25 ± 0.21^b^	21.97 ± 2.12^b^	22.20 ± 0.71^b^
Chewiness (mj)	56.40 ± 4.30^b^	63.10 ± 4.00^a^	53.80 ± 0.75^b^	34.26 ± 2.98^c^	30.10 ± 1.88^c^	27.95 ± 1.00^c^	30.50 ± 0.48^c^
Adhesiveness (mj)	1.29 ± 0.05^ab^	0.75 ± 0.14^d^	0.76 ± 0.00^d^	0.97 ± 0.07^cd^	1.05 ± 0.10^bc^	1.53 ± 0.21^a^	1.53 ± 0.09^a^

*Note*: Mean values in the same row with different letters are significantly different (*p* < .05).

### 3.4 Sensory properties of SPLSNs

As shown in Table 3, the sensory scores of SPLSNs increased as the particle size of SPLs powder decreased. The total scores for CK, CP, SG‐3 to SG‐15 were 89.20, 86.21, 88.40, 89.24, 90.61, 90.21, and 90.70, respectively. The color of the SPLSNs was improved by the color of the sweet potato leaves. In particular, the SG‐12 and SG‐15 samples had a uniform color, and the color for those samples was close to that of the sweet potato leaves.

### 3.5 Textural properties of SPLSNs

Table 3 shows the TPA parameters of the cooked SPLSNs. The hardness of SPLSNs increased as the milling time increased. In particular, the highest hardness in the SG‐15 sample was probably associated with its lowest WHC. The quantity of starch granules released in the cooking water and adhered to the surface of the cooked noodles is referred to as adhesiveness. As a result, greater adhesiveness is associated with higher cooking loss. However, in this study, noodles of SG‐12 and SG‐15 had the highest adhesiveness (0.15% and 0.14% cooking loss, respectively), while noodles of CP and SG‐3 had the lowest adhesiveness (0.23% and 0.18% cooking loss, respectively) (*p* < .05). The chewiness values of SPLSNs varied, with SG‐6, SG‐9, SG‐12, and SG‐15 having the lower values, while CK, CP, and SG‐3 had the higher values (*p* < .05). The hardness, cohesiveness, and springiness are used to determine the chewiness. Hence, changes in those values have a direct impact on chewiness. The values of cohesiveness and springiness decreased as the particle size of SPLs powder reduced. There was a difference in cohesiveness or springiness between CP and other treatments (*p* < .05). This would attribute to the SPLs powder, which affected the aggregation among starches. Since chewiness is associated with solid foods, gumminess was not considered as a parameter in the TPA of starch noodles. Odabas et al. (2022) reported that the hardness and chewiness of gluten‐free noodles increased with an increase in the percentages of quinoa and yellow lentil flour and peaked at the highest levels of incorporation (50%). Liu et al. (2021) found that low‐amylose rice starch significantly increased the tensile strength and hardness of potato starch noodles, whereas waxy rice starch had the reverse effect. Waliullah et al. (2021) reported that the textural properties of starch noodles could be improved by ultrafine sweet potato residues. Odabas et al. (2022) discovered that the hardness of gluten‐free noodles ranged from 7.1 to 14.0 N, with no apparent relationship to the amount of yellow lentil flour.

### 3.6 Long‐range ordered structure

**FIGURE 1:**
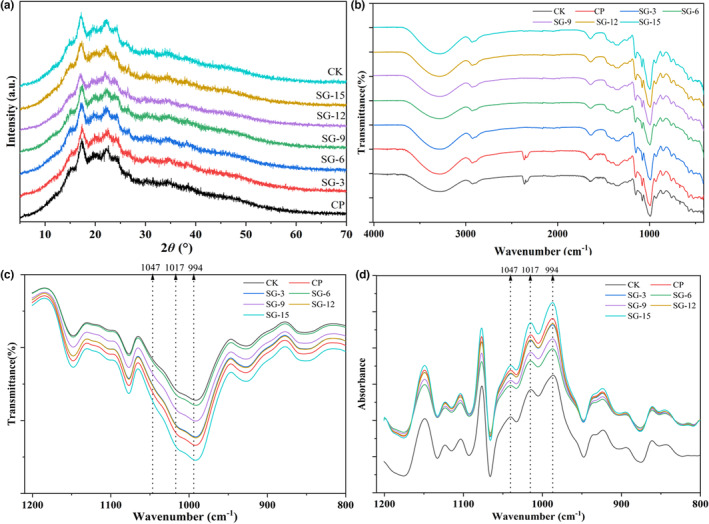
XRD diffractograms and FT‐IR spectra of dried SPLs sweet potato starch noodles. (a) XRD diffraction; (b) FT‐IR spectra of samples; (c): infrared absorption peaks in the range of 1200 to 900 cm^−1^; (d) deconvoluted FT‐IR spectra of samples between 1200 and 900 cm^−1^.

The X‐ray diffraction (XRD) was used to determine the starch's long‐range molecular order. The diffraction pattern of sweet potato starch (SPS) is a classic B‐type pattern, with the biggest diffraction peak at 17° (2*θ*) and a few smaller peaks at around 5.6°, 15°, 22°, and 24° (Yang, Zhang, et al., 2021). Due to the gelatinization and retrogradation, SPLSNs had a more diffuse XRD diffraction pattern (but still a B‐type crystalline pattern) with peaks at approximately 17° and 22° (Figure 1a and Table 4). The crystallinity of SPLSNs ranged from 14.19% to 28.85% (Table 1). A difference was found in crystallinity between SG‐12 (or SG‐15) and CP (*p* < .05). It showed that the less ordered or stable recrystallized structure was obtained (Yang, Dhital, et al., 2021; Yang, Zhang, et al., 2021). This may be due to the fact that SPLs powder with a smaller size would be easy to release more polyphenols, which limited the chain fluidity and prevented the rearrangement of starch molecules. Curcumin, tea polyphenols, and lycopene were found to damage the crystal forms and interfere with starch crystallization (2022).

**TABLE 4:** Crystallinity and FT‐IR deconvolution results of SPLs sweet potato starch noodles.

Parameter	XRD				FT‐IR			
	Diffraction peaks at 2θ (°)		Crystallinity(%)	Pattern	Shift_3290_	Shift_2930_	*R*_1047/1017_	*R*_1017/994_
CK	17.22	22.32	28.69 ± 0.94^ab^	B	3278.21 ± 1.07^b^	2926.65 ± 0.23^ab^	1.14 ± 0.05^a^	0.39 ± 0.01^ce^
CP	17.32	22.60	28.85 ± 0.44^ab^	B	3275.92 ± 0.27^c^	2925.84 ± 0.66^bc^	0.96 ± 0.03^cd^	0.41 ± 0.02^b^
SG‐3	17.40	22.56	28.58 ± 1.32^b^	B	3274.68 ± 0.71^c^	2925.71 ± 0.32^c^	0.92 ± 0.03^d^	0.40 ± 0.01^bc^
SG‐6	17.40	22.08	29.99 ± 0.61^a^	B	3272.75 ± 0.80^d^	2926.09 ± 0.35^abc^	0.98 ± 0.06^bc^	0.40 ± 0.01^bc^
SG‐9	17.44	22.36	29.92 ± 0.24^ab^	B	3287.00 ± 1.22^a^	2926.27 ± 0.35^abc^	1.02 ± 0.03^b^	0.40 ± 0.01^bc^
SG‐12	17.16	22.86	20.77 ± 0.24^c^	B	3277.90 ± 0.52^b^	2926.88 ± 0.19^a^	0.95 ± 0.04^cd^	0.40 ± 0.01^bcd^
SG‐15	17.38	22.38	14.19 ± 0.52^d^	B	3278.21 ± 0.03^b^	2876.46 ± 0.09^abc^	0.81 ± 0.06^e^	0.43 ± 0.00^a^

*Note*: Mean values in the same column with different letters are significantly different (*p* < 05).

### 3.7 Short‐range molecular order of starch

The short‐range order of starch can be investigated using FT‐IR. As shown in Figure 1b, all SPLSNs displayed typical carbohydrate absorption peaks between 4000 and 500 cm^−1^. Each sample showed an intense and broad infrared absorption peak between 3700 and 3000 cm^−1^, corresponding to the starch O‐H stretching vibration. The absorption peak at 2930 cm^−1^ was attributed to the vibrations of CH_2_ (Li et al., 2016; Yang, Dhital, et al., 2021; Yang, Zhang, et al., 2021). Pure sweet potato starch noodles had a ‐OH peak with a wavenumber of 3278.21 cm^−1^, but the peaks in SPLSNs samples for CP, SG‐3, SG‐6, SG‐9, SG‐12, and SG‐15 were 3275.92, 3274.68, 3272.75, 3287.00, 3277.90, and 3278.21 cm^−1^, respectively (Table 4). Starch noodles had greater wavenumber shifts and less flat ‐OH peak bands than natural potato starch (3284 cm^−1^), which showed that the hydrogen bonds between molecules were weaker and lower (Liu et al., 2018). It was determined that the H‐bonding connection between the molecules of SG‐6 to SG‐15 was displaced less than that of CK, and this was attributable to the less stable or organized crystalline structure generated during the recrystallization.

Figure 1c,d show the short‐range ordered structure of SPLSNs by further deconvoluting the distinctive bands between 1200 and 900 cm^−1^. Due to its sensitivity to C‐C, C‐OH, and C‐H stretching vibrations, this absorption zone has been discovered as a fingerprint absorption region for starch‐based samples and may indicate changes in the conformation of starch polymers. The molecular order and crystallinity of starch polymers are shown to be connected with the peaks at 1047 and 994 cm^−1^, respectively, while the band intensity at 1017 cm^−1^ represents the disordered or amorphous phase of starch. Quantifying the degree of order in the starch and describing the internal alterations in the double helix can be done using the *R*_1047/1017_ and *R*_1017/994_. In Figure 1d, the absorption band at 1047 cm^−1^ displayed a narrow and evident peak in each sample. *R*_1047/1017_ and *R*_1017/994_ of pure starch noodles were 1.14 and 0.39. When SPLs powder was added, *R*_1047/1017_ value markedly decreased from 1.14 to 0.81, while the *R*_1017/996_ value increased from 0.39 to 0.43. It indicated that SPLSNs have a lower crystalline region and fewer ordered double helices, supported by the above‐mentioned XRD analysis (Table 4). This lower ordered structure in SPLSNs might be attributed to the particle and substance released from leaves which could destroy crystallization processing and prevent the mobility of double helices for better alignment. Yang, Dhital, et al. (2021) reported that wet starch noodles had a larger proportion of crystalline area and more complete crystallites than dried samples. Yang, Zhang, et al. (2021) found that with the extension of storage time, the crystallinity, double helix, and water mobility decreased, resulting in the disorganization of the supramolecular structure.

### 3.8 Morphological characteristics of SPLSNs

**FIGURE 2:**
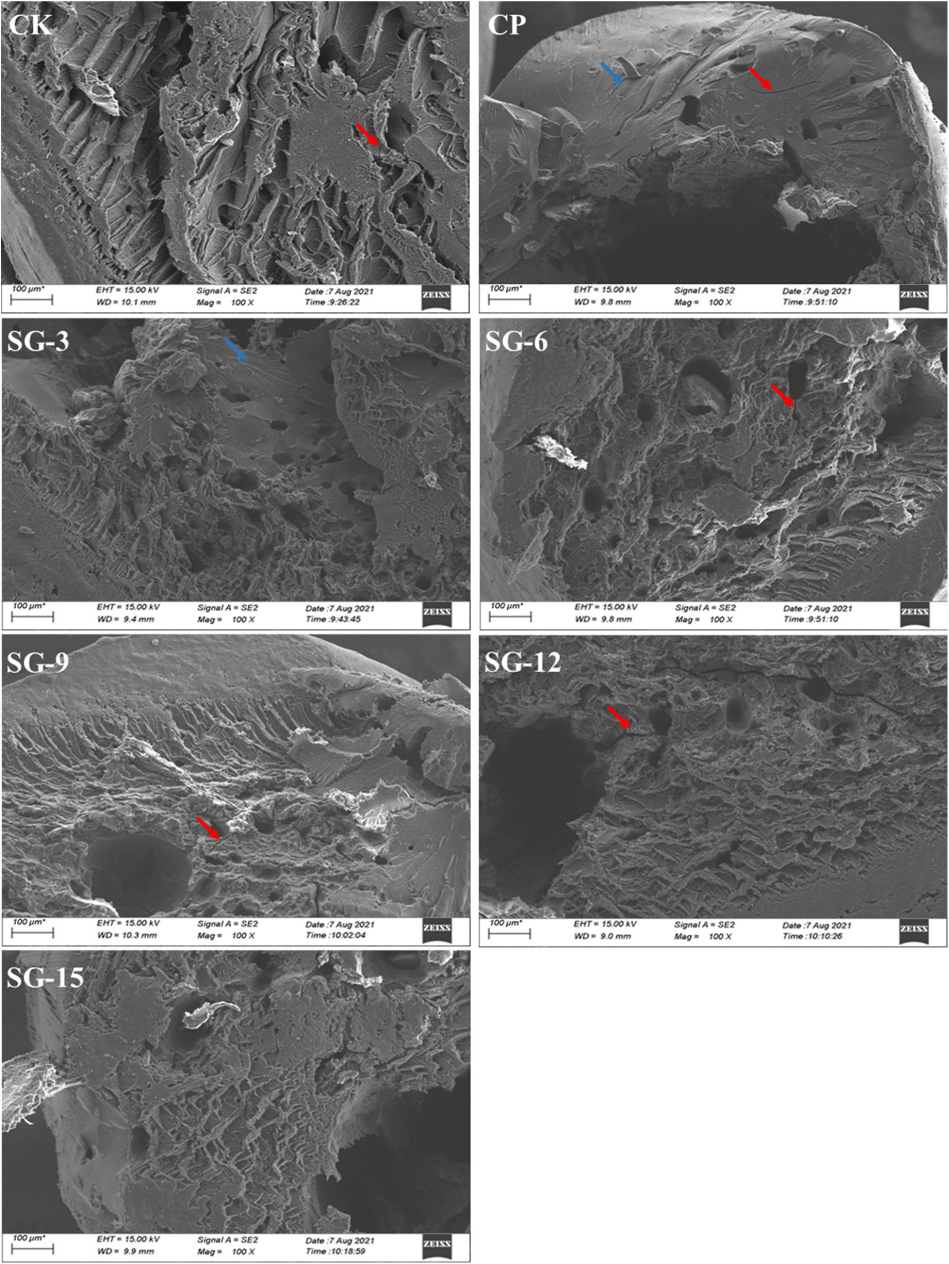
Microstructure of cross section of dried SPLs sweet potato starch noodles.

SEM was used to examine microimages of cross sections of the dried SPLSNs, and the morphology is shown in Figure 2. The process used to make the noodles entirely damaged the native potato starch granules, which had flat, smooth surfaces, and oval and round forms. A distinctive block and layered strip structure (blue arrows) was formed. This could be due to the amylose leaching, the loss of the amylopectin crystalline zone, and recrystallization. All cross‐sectional images of dried SPLSNs showed a compact glassy state with air bubbles and small inside pores with fissures (red arrows). Compared with the CP, the more compact and less cracks and holes were found with decreasing the particle size. A similar phenomenon has also occurred in other noodles ( Li et al., 2022; Odabas et al., 2022; Wu et al., 2015; Xiang et al., 2018; Yang, Dhital, et al., 2021; Yang, Zhang, et al., 2021).

## 4 CONCLUSION

In this study, compared with coarse powder, the superfine grinding powder of SPLs, with a mean particle size of 26.11 μm, showed smaller size, bulk and tapped density, repose and slide angle, WHC, OBC, and SC, as well as higher specific surface area and WSI. In order to enhance the nutrients of SPSN, the SPLs powder was used as a starch substitute to make starch noodles. Results showed that SPLs powder changed the CL, SI, texture, and sensory properties of SPLSNs. XRD showed that the crystal structure of SPLSNs was destroyed as the crystallinity decreased from 28.85% to 14.19%. FT‐IR displayed that the SPLSNs had less proportion of crystalline region (*R*_1047/1017_ from 0.96 to 0.81, *R*_1017/994_ from 0.41 to 0.43). SEM demonstrated that fewer pores and cracks in cross section were found in SPLSNs as the particle size reduced. In the future, the effect of different levels (0%–5%–10%–155–20%) of SPLs powder on the quality of SPSN will be investigated.

## AUTHOR CONTRIBUTIONS

**Guanghui Li:** Conceptualization (equal); formal analysis (equal); investigation (equal); methodology (equal); writing – original draft (lead). **Xueli Gao:** Project administration (equal); supervision (equal); validation (equal). **Yonghui Wang:** Investigation (equal); writing – original draft (equal). **Shenghua He:** Funding acquisition (supporting); supervision (equal); writing – review and editing (equal). **Weiyun Guo:** Funding acquisition (equal); project administration (equal); supervision (equal); validation (equal). **Jihong Huang:** Supervision (equal); validation (equal).

## FUNDING INFORMATION

This work was supported, in part, by the Program for the Major Science and Technology Projects in Henan Province, China (ID No. 201300110300), Key Scientific Research Projects of Universities in Henan Province (ID No. 22B550017), and Special Funds for Talents from Xuchang University.

## CONFLICT OF INTEREST STATEMENT

The authors have no conflict of interest regarding the publication of the paper.

## ETHICS STATEMENT

This study does not involve any human or animal testing.

## CONSENT FOR PUBLICATION

All listed authors have read the final manuscript and provided consent for publication.

## Data Availability

The data that support the findings of this study are available on request from the corresponding author.
